# Risk factors for bone cement leakage in percutaneous vertebroplasty and its prevention

**DOI:** 10.12669/pjms.42.3.13396

**Published:** 2026-03

**Authors:** Chenyang Jiang, Jun Miao

**Affiliations:** 1 **Chenyang Jiang** Department of Orthopaedics, Clinical School/College of Orthopaedics, Tianjin Medical University, Tianjin 300070, China; 2 **Jun Miao** Department of Spinal Surgery, Tianjin University Tianjin Hospital, Tianjin 300110, China

**Keywords:** Bone cement leakage, Percutaneous vertebroplasty, Prevention strategy, Risk factors

## Abstract

**Objectives:**

To analyze the risk factors for bone cement leakage during percutaneous vertebroplasty (PVP) and establish a predictive model to guide clinical prevention.

**Methodology:**

This retrospective study analyzed 120 patients with osteoporotic vertebral compression fractures (OVCF) who underwent PVP between June 2018 to June 2022. Based on postoperative imaging, patients were divided into leakage group (72 vertebral bodies) and non-leakage group (84 vertebral bodies). Univariate and multivariate logistic regression analyses were performed on 15 clinical and radiographic variables. The predictive performance was evaluated using receiver operating characteristic (ROC) curve analysis.

**Results:**

This study included 120 patients, including 69 males and 51 females, with a mean age of 70.97 ± 5.04 years (range: 62–83 years),the analysis unit was the vertebral body, as some patients underwent multi-level PVP procedures, with the involvement of 156 vertebral bodies totally, 72 vertebral bodies had bone cement leakage and 84 vertebral bodies had no leakage, with a total leakage rate of 46.15% (72/156). Bone density (OR=4.953, P=0.001), cortical defect (OR=3.494, P=0.005), and bone cement injection volume (OR=2.885, P=0.016) were identified as independent risk factors for cement leakage. The combined predictive model incorporating these three factors demonstrated the highest diagnostic accuracy, with an area under the curve (AUC) of 0.774 (P<0.001).

**Conclusion:**

Low bone density, presence of cortical defects, and excessive cement volume independently increase leakage risk during PVP. Preoperative assessment and individualized cement injection strategies based on these factors may help reduce complication rates.

## INTRODUCTION

There is an increasing prevalence of osteoporosis in the elderly with the accelerating pace of aging and the increase in the number of the elderly in China. Osteoporosis may result in the decrease in bone density and bone strength, triggering an increase in the incidence of osteoporotic vertebral compression fractures (OVCF) yearly.^1^ Clinically, patients suffering from OVCF may usually experience lower back pain, spinal deformities, and limited mobility. Patients bedridden due to pain can further develop secondary cardiovascular and pulmonary dysfunction, muscle weakness, etc., which seriously compromise their quality of life.^2^ Noticeably, minimally invasive spinal techniques have continuously developed and advanced since the application of bone cement in treating vertebral hemangioma was reported in 1987.^3^

Nowadays, percutaneous vertebroplasty (PVP) has gained popularity in the treatment of OVCF due to its advantages of less trauma, faster pain relief, and better postoperative recovery compared to open surgery.^4^ However, with the widespread clinical application, PVP-related complications, bone cement leakage in particular, have also been extensively concerned. There is currently no unified standard for the volume of bone cement injected during PVP. In case of insufficient quantity of bone cement injected, it cannot solidify the vertebral body and stabilize the spine, accompanied by poor postoperative pain relief; while increasing the volume of bone cement can promote its widespread dispersion within the vertebral body, resulting in better surgical outcomes.

Nevertheless, improper or excessive injection of bone cement can increase the risk of leakage. In mild cases, there may be significant clinical discomfort; while some patients may experience pulmonary embolism and nerve damage, and even paraplegia or death in severe cases.^5^ Therefore, to systematically identify the independent risk factors for bone cement leakage during PVP and establish a clinically applicable predictive model, this study retrospectively analyzed perioperative data from OVCF patients. The findings aim to provide evidence-based guidance for preoperative risk stratification and intraoperative decision-making, ultimately reducing leakage-related complications and improving surgical safety.

## METHODOLOGY

A retrospective analysis was conducted on the data of OVCF patients undergoing PVP in Baoding No.1 Hospital from June 2018 to June 2022. The enrolled patients were divided into a leakage group and a non-leakage group based on the presence of postoperative bone cement leakage. Although data collection spanned 2018–2022, the completion of comprehensive data verification, extended outcome evaluation, and multicenter collaboration delayed manuscript preparation. All data reflect standard PVP practices that remain clinically relevant. Patients were categorized based on postoperative imaging findings: vertebral bodies exhibiting cement leakage outside the vertebral confines were assigned to the leakage group, while those without leakage were assigned to the non-leakage group. The unit of analysis was the vertebral body, as some patients underwent multi-level PVP.

### Ethical approval:

The study was approved by the Institutional Ethics Committee of Baoding No.1 Hospital (No.:2021326; Date: March 26, 2021), and written informed consent was obtained from all participants.

### Inclusion criteria:

- Patients diagnosed with fresh OVCF through imaging examination and treated with PVP.
- Individuals over 50 years old.
- Patients with complete medical records and imaging data.

### Exclusion criteria:

- Patients with pathological fracture.
- Patients with vertebral deformities and lesions adjacent to the injured vertebrae, such as old vertebral compression fractures.
- Individuals with a history of PVP.
- Patients with incomplete medical records and imaging data.

### Surgical approach and determination for bone cement leakage:

All PVP procedures were completed by the same group of physicians with the title of deputy chief physician or above in our hospital. Under local anesthesia, patients in the prone position, with chest and abdomen suspended, underwent surgery under the condition of vital sign monitoring throughout the entire process. X-ray fluoroscopy was employed to mark the projection points on the surface of the injured vertebral pedicle, and after positioning, the C-arm imaging system was used to guide the unilateral or bilateral puncture using puncture needle through the transpedicular approach.

The puncture was stopped when the tip of the puncture needle was located at the junction of the anterior and middle one-third of the vertebral body. Following the mixing, the bone cement in the wire-drawing stage was injected into the vertebral body using a bone cement injector. Furthermore, the diffusion of bone cement was dynamically observed under C-arm fluoroscopy. The injection was terminated when the bone cement reached the edge of the vertebral body (i.e. the cortical bone). Then, with the puncture needle pulled out, the incision was wrapped with dressing, and the surgery ended. The presence of bone cement leakage was determined when bone cement was located outside the vertebral body with intraoperative C-arm fluoroscopy or postoperative X-ray plain scan. Simultaneously, CT and/or MRI examinations were adopted to determine the leakage path and evaluate the condition of the spinal canal.

### Outcome measures:

Data of the enrolled patients was collected from the Medical Record System of this hospital, including gender, age, body mass index (BMI), bone density, trauma history, underlying diseases, preoperative Visual Analogue Scale (VAS) score, Oswestry Disability Index (ODI) score, fracture segment, degree of vertebral compression, intravertebral cleft, cortical defect, number of surgical vertebrae, unilateral or bilateral puncture, and bone cement injection volume. All these indicators were statistically analyzed to determine whether they were risk factors for bone cement leakage in PVP. Among these, the degree of pain was evaluated using the VAS scale, while daily activity was assessed using the ODI. The present analysis focused on perioperative cement leakage as the primary endpoint. Parameters such as limb shortening, gait analysis, and postoperative range of motion were not systematically evaluated, and long-term functional follow-up was beyond the scope of this retrospective review.

### Statistical analyses:

SPSS 22.0 software was used for data processing and statistical analysis. Counting data was expressed as percentages (%), while measurement data was described as *x̅* ± *s*, with corresponding comparison performed using the c^2^ test, and *t*-test, respectively. Following univariate analysis, variables with statistical significance (*P*<0.05) were selected as independent variables for multivariate logistic regression analysis to screen for independent risk factors for bone cement leakage in PVP. Meanwhile, the receiver operating characteristic (ROC) curve was plotted to compare the area under the curve (AUC) of various factors. The predictive value of bone cement leakage was evaluated using sensitivity, specificity, and AUC. *P*<0.05 meant that the difference was statistically significant.

## RESULTS

This study included 120 patients (69 males, 51 females) diagnosed with OVCF who underwent PVP treatment, with a mean age of 70.97 ± 5.04 years (range: 62–83 years). The analysis unit was the vertebral body, as some patients underwent multi-level PVP procedures, with the involvement of 156 vertebral bodies totally, 72 vertebral bodies had bone cement leakage and 84 vertebral bodies had no leakage, with a total leakage rate of 46.15% (72/156). In addition, all patients did not experience serious complications such as pulmonary embolism or nerve damage. A univariate analysis was performed on 15 independent variables including patient age, gender, BMI, VAS score, etc. As presented in Table-I, the results showed that bone density, trauma history, underlying diseases, degree of vertebral compression, intravertebral cleft, cortical defect, and bone cement injection volume might be potential risk factors leading to the occurrence of bone cement leakage in PVP (all *P*<0.05).

**Table-I:** Univariate analysis of factors affecting bone cement leakage.

Factors	Leakage group	Non-leakage group	t/*χ*^2^	P
Gender			0.020	0.888
Male	32 (55.17)	35 (56.45)		
Female	26 (44.83)	27 (43.55)		
Age (years)	70.60±5.15	71.31±4.94	0.763	0.447
BMI	23.40±3.21	22.97±3.80	0.666	0.507
Bone density	4.02±0.91	3.23±0.90	4.786	<0.001
Trauma history			7.427	0.006
With	35 (60.34)	22 (35.48)		
Without	23 (39.66)	40 (64.52)		
Underlying diseases			5.670	0.017
With	36 (62.07)	25 (40.32)		
Without	22 (37.93)	37 (59.68)		
Preoperative VAS score	6.86±0.69	6.97±0.75	0.806	0.422
ODI score	76.72±7.04	75.81±7.80	0.675	0.501
Fracture segment			0.049	0.976
Thoracic	12 (16.67)	15 (17.86)		
Thoracolumbar	43 (59.72)	50 (59.52)		
Lumbar	17 (23.61)	19 (22.62)		
Degree of vertebral compression			6.529	0.038
≤25%	13 (18.06)	20 (23.81)		
26%-50%	29 (40.28)	45 (53.57)		
>50%	30 (41.66)	19 (22.62)		
Intravertebral cleft			8.668	0.003
With	47 (65.28)	35 (41.67)		
Without	25 (34.72)	49 (58.33)		
Cortical defect			7.603	0.006
With	27 (37.50)	15 (17.86)		
Without	45 (62.50)	69 (82.14)		
Number of surgical vertebrae	1.74±0.58	1.76±0.59	0.156	0.876
Unilateral/bilateral puncture			0.707	0.401
Unilateral	40 (55.56)	41 (48.81)		
Bilateral	32 (44.44)	43 (51.19)		
Bone cement injection volume	6.10±1.31	5.48±1.55	2.677	0.008

Factors with statistically significant differences (all *P*<0.05) in the univariate analysis were incorporated into multivariate logistic regression analysis. Corresponding results, as summarized in Table-II, revealed that bone density, cortical defect, and bone cement injection volume were independent risk factors affecting bone cement leakage in PVP (all *P*<0.05); while trauma history, underlying diseases, degree of vertebral compression, and intravertebral cleft were not independent risk factors for bone cement leakage in PVP (all *P*>0.05).

**Table-II:** Multivariate logistic regression analysis of factors affecting bone cement leakage during PVP.

Variables	Regression coefficient	Standard error	Wald*χ*^2^	P	OR( 95%CI)
Bone density	1.600	0.483	10.973	0.001	4.953(1.922-12.766)
Cortical defect	1.251	0.450	7.713	0.005	3.494(1.445-8.449)
Bone cement injection volume	1.059	0.442	5.750	0.016	2.885(1.214-6.858)

**Fig.1:**
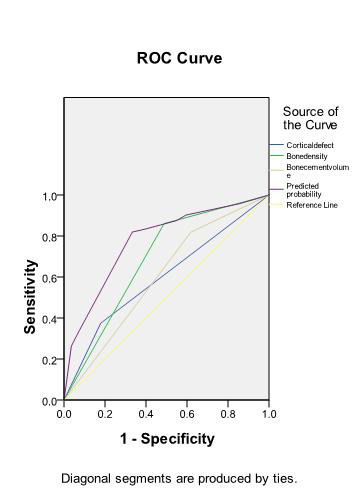
ROC curves of various models predicting bone cement leakage.

The ROC curve for predicting bone cement leakage in PVP was plotted based on the results of logistics analysis. Consequently, the combined model established based on the risk factors of bone cement leakage exhibited relatively higher predictive performance, followed by the model using bone density, and the model using cortical bone defect alone displayed the worst predictive performance (Table-III and Fig.1).

**Table-III:** ROC curve-based prediction efficiency of bone cement leakage during PVP.

Variables	AUC	SE	P	95%CI
Bone density	0.687	0.043	0.000	0.603-0.770
Cortical defect	0.598	0.046	0.035	0.508-0.688
Bone cement injection volume	0.600	0.045	0.031	0.512-0.689
Combined model	0.774	0.038	0.000	0.699-0.848

## DISCUSSION

Based on the clinical data analysis of 120 patients with osteoporotic vertebral compression fractures (OVCF), this study systematically identified independent risk factors for cement leakage during percutaneous vertebroplasty (PVP) and constructed a combined prediction model with good predictive performance. The research results showed that decreased bone mineral density, cortical bone defects in the vertebral body, and excessive cement injection volume were three independent risk factors for cement leakage. The combined prediction model constructed based on these three factors exhibited optimal diagnostic performance (AUC=0.774), providing a quantitative tool for preoperative risk assessment in clinical practice. This study found that bone mineral density (T-score) is the strongest independent factor in predicting cement leakage (OR=4.953). This result is highly consistent with multiple research conclusions, indicating that the higher the degree of osteoporosis, the higher the risk of cement leakage. The potential mechanisms may include:

- osteoporosis leads to sparse bone trabeculae and increased porosity within the vertebral body, forming microscopic channels that allow cement to easily diffuse along unexpected pathways;^6^
- decreased bone strength makes the vertebral body more prone to microfractures during injection, forming new leakage pathways;^7^
- low-density bone has weaker “constraint” on cement, making cement more prone to paravertebral venous leakage.^8^ Clinically, patients with a bone mineral density T-score < -3.0 should be considered high-risk individuals. In addition to conventional anti-osteoporosis treatment, intraoperative measures such as using high-viscosity cement, slowing down the injection rate, and injecting during the “dough phase” rather than the “string phase” to reduce fluidity are recommended.

This study confirms that vertebral cortical bone defects, especially posterior wall defects, are an independent risk factor for cement leakage (OR=3.494). This is consistent with the viewpoint that “cortical integrity is the key pathway for leakage”.^9^ When fractures involve the posterior wall of the vertebral body, cement can directly spill into the spinal canal, compressing the spinal cord or nerve roots, leading to neurological impairment, and in severe cases, even paralysis.^10^,^11^ Therefore, thin-layer CT examination must be performed before surgery to accurately assess cortical integrity. For patients with clear posterior wall defects, PVP should be carefully selected, or consideration should be given to switching to posterior internal fixation combined with limited vertebroplasty. If PVP is still performed, the puncture needle should be kept away from the defect area, and the injection should be carried out using a “low pressure, slow push, intermittent” strategy under close monitoring under fluoroscopy.^12^

In this study, for every one mL increase in cement injection volume, the risk of leakage increased by approximately 2.9 times (OR=2.885). This is consistent with some research findings that excessive cement can increase intravertebral pressure,^13^,^14^ forcing the cement to overflow through venous sinuses or fracture lines. However, insufficient cement volume (such as <3 mL) may lead to weak mechanical support of the vertebral body, increasing the risk of postoperative refracture.^15^ Therefore, we advocate the principle of “individualized appropriate injection”: a comprehensive judgment based on vertebral volume, degree of compression, bone density, and fracture type. Generally speaking, an injection volume of 3-5 mL per vertebral body is appropriate, and blindly pursuing complete vertebral body filling should be avoided. For patients with long-segment fractures or significant vertebral body deformities, multiple injections or bilateral punctures may be considered to improve the uniformity of cement distribution.^16^

Compared with previous studies,^17^,^18^ this model systematically integrates preoperative assessment variables (bone density, cortical defects) and intraoperative controllable variables (cement volume); provides a quantifiable risk prediction tool (AUC=0.774); emphasizes the importance of preoperative CT assessment of cortical defects, providing a basis for surgical plan selection. Meanwhile, this study suggests that in static assessment, anatomical factors of cortical defects may have more predictive value.

Based on the results of this study, we recommend routine bone density measurement and vertebral CT assessment for all OVCF patients undergoing PVP, to identify patients at high risk due to both “low bone density” and “cortical defect”.^19^ For high-risk patients, it is recommended to use high-viscosity cement, control the cement volume per vertebra (≤4 mL), and prioritize bilateral puncture. If cortical defects are confirmed, intraoperative balloon kyphoplasty (PKP) or combined posterior fixation should be considered.^20^ Additionally, for high-risk patients, postoperative monitoring time should be extended, and close observation for neurological symptoms or respiratory distress should be conducted. Postoperative CT reexamination should be performed if necessary.

### Limitations:

It includes its retrospective design, modest sample size, and single-center origin. Functional outcomes such as gait improvement or range of motion were not assessed, and long-term follow-up was not performed.

## CONCLUSIONS

This study identifies bone density, cortical defect, and bone cement injection volume as independent risk factors for bone cement leakage in OVCF patients undergoing PVP. Findings in our study suggest that for OVCF patients scheduled to receive PVP, it is critical to adopt detailed preoperative examination and enhanced safety assessment, as well as precise and standardized operation intraoperatively. Comprehensive bone cement injection strategies tailored to patients’ condition should be developed to minimize the occurrence of bone cement leakage.

### Authors’ Contributions:

**CJ:** Conceived and designed the study, performed statistical analysis, drafted and critically revised the manuscript, and is responsible for the overall content and integrity of the work. Conducted literature review, assisted in data collection and preliminary drafting.

**JM:** Collected clinical data, data interpretation. Critical Review.

All authors have reviewed and approved the final version of the manuscript.
